# 

**Published:** 1886-08

**Authors:** 


Whole Number, 319. AUGUST, 1886. Vol. LIII. Number 2.

LIBRARY
INDEXED
S.G.O.

### THE CHICAGO MEDICAL JOURNAL AND EXAMINER

EDITORS:

S. J. JONES, M.D., LL.D.

W. W. JAGGARD, A.M., M.D. HAROLD N. MOYER, M.D.

PUBLISHED MONTHLY BY

THE CLARK & LONGLEY CO.,

Under direction of

THE CHICAGO MEDICAL PRESS ASSOCIATION,

308-316 DEARBORN STREET.

